# Safety and effectiveness of colistin compared with tobramycin for multi-drug resistant *Acinetobacter baumannii *infections

**DOI:** 10.1186/1471-2334-9-26

**Published:** 2009-03-09

**Authors:** Ronald Gounden, Colleen Bamford, Richard van Zyl-Smit, Karen Cohen, Gary Maartens

**Affiliations:** 1 Department of medicine, division of clinical pharmacology, University of Cape Town, Cape Town, South Africa; 2 Department of clinical laboratory sciences, University of Cape Town, Cape Town, South Africa; 3 Department of medicine, division of respiratory medicine University of Cape Town, Cape Town, South Africa

## Abstract

**Background:**

Nosocomial infections due to multi-drug resistant *Acinetobacter baumannii *are often treated with colistin, but there are few data comparing its safety and efficacy with other antimicrobials.

**Methods:**

A retrospective cohort study of patients treated with colistin or tobramycin for *A. baumannii *infections in intensive care units (ICUs) at Groote Schuur hospital. Colistin was used for *A. baumannii *isolates which were resistant to all other available antimicrobials. In the tobramycin group, 53% of the isolates were only susceptible to tobramycin and colistin. We assessed ICU mortality, nephrotoxicity and time to the first negative culture.

**Results:**

32 patients, with similar admission APACHE scores and serum creatinine, were treated with each antimicrobial. There were no significant differences between the colistin and tobramycin groups in ICU mortality (p = 0.54), nephrotoxicity (p = 0.67), change in creatinine from baseline to highest subsequent value (p = 0.11) and time to microbiological clearance (p = 0.75). The hazard ratio for total in-hospital survival in patients treated with colistin compared to tobramycin was 0.43 (95% CI 0.19 to 0.99).

**Conclusion:**

Our study suggests that colistin and tobramycin have similar risks of nephrotoxicity and are equally efficacious. Colistin is an acceptable antibiotic for the treatment of *A. baumanii *infections when the organism is resistant to other available antimicrobials.

## Background

Colistin (polymyxin E) has been available since 1959. Since the 1970's, other classes of antimicrobials, such as the aminoglycosides or carbapenems were favoured for gram-negative infections [1] because of concerns regarding colistin's nephrotoxicity. However, the worldwide emergence of multi-drug resistant nosocomial gram-negative pathogens over the last decade has resulted in the increased use of colistin as last resort therapy [2,3].

Dosing recommendations for colistin vary considerably [4], and, unlike aminoglycosides, therapeutic monitoring of colistin plasma concentrations is not readily available. Nephrotoxicity has previously been shown to occur in 14.3 to 24% of patients treated with colistin in intensive care units (ICUs) [5-7]. However, sepsis, hypotension and the use of other nephrotoxic drugs may have contributed to the impairment of renal function observed in these studies.

In the intensive care units (ICUs) at Groote Schuur Hospital, multi-drug resistant *Acinetobacter baumannii *infections have become common. **Multi-drug resistant organisms **are defined as resistant to three or more antimicrobial classes normally used for the treatment of infections [8]. The aminoglycoside tobramycin is the antimicrobial most commonly prescribed at Groote Schuur for susceptible *A. baumannii *infections but resistance to tobramycin occurs commonly (41% of isolates in 2006). All *A. baumannii *isolates at Groote Schuur Hospital remain susceptible to colistin, which is prescribed when the organism is resistant to all other available antimicrobials. Tobramycin or colistin are used as monotherapy for *Acinetobacter baumannii *infections at Groote Schuur Hospital. The aim of our study was to compare the safety and effectiveness of **colistin versus tobramycin **in ICU patients with *A. baumannii *infections.

## Methods

A retrospective chart review of patients in the ICUs of Groote Schuur Hospital was conducted. Groote Schuur Hospital is an 867-bed tertiary referral centre in Cape Town, South Africa, with 56 ICU beds (respiratory, general surgery and neurosurgery). All patients treated with colistin for *A. baumannii *infections in Groote Schuur Hospital ICUs between January 2003 and December 2005 were identified from pharmacy records.

**Figure 1:**
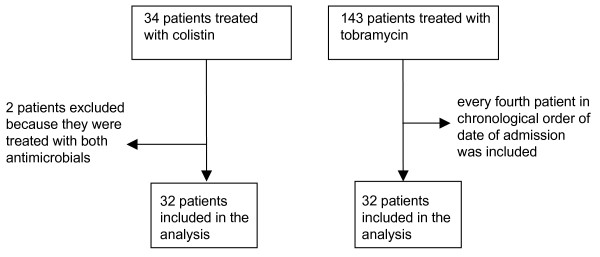
**Study profile**.

Figure 1 shows the study profile. Pharmacy records identified 34 patients who had been treated with colistin and 143 with tobramycin for ICU-acquired *A. baumannii *infections. Two patients had received both colistin and tobramycin during their ICU stay and were excluded from the analysis. A total of 64 medical records were evaluated. Every fourth patient who received tobramycin, in order of date of admission to ICU, was included.

The patient's health status on admission as measured by the Acute Physiology and Chronic Health Evaluation II (APACHE) score, clinical course in ICU and other antimicrobials used were captured using a standardised data collection form. Laboratory results were obtained from the Groote Schuur National Health Laboratory Services electronic database.

Acinetobacter baumannii was tested for susceptibility to piperacillin-tazobactam, colistin, ceftazidime, cefepime, imipenem, meropenem, gentamicin, amikacin, tobramycin and ofloxacin/ciprofloxacin and colistin. For all antibiotics except colistin, susceptibility testing was performed using the Kirby-Bauer disc diffusion method on Mueller Hinton agar (Oxoid). Minimum inhibitory concentrations for colistin were determined by E-test (AB Biodisk, Solna, Sweden) according to the manufacturer's instructions. Interpretative criteria according to Clinical Laboratory Standards Institute guidelines were used.

The following were regarded as criteria to support the use of antimicrobials for *A baumannii*: positive blood culture for *A. baumannii *or culture of *A. baumannii *from other sites associated with clinical signs of infection (a temperature greater than 38°C, white cell count greater than 12×10^9^/L, new chest x-ray infiltrates, the presence of purulent sputum/tracheal aspirate or the necessity for inotropic support) documented within 48 hours of commencing the antimicrobial [9,10].

At Groote Schuur Hospital, colistin (Colimycine^® ^Aventis, Bellon, France) is administered at a dose of 2 million units 8 hourly in patients with normal renal function. The manufacturers recommend 50 000 IU/kg/day in 2–3 divided doses [11], which for a 70 kg adult amounts to 1.2 million IU 8 hourly. Our practise is based on studies that have used up to 3 million units 8 hourly [5,6], with toxicity reported at these dosing levels not markedly increased compared with the lower recommended doses.

Colistin dosage was adjusted for renal failure as follows: creatinine clearance 50–90 ml/min 2 million units 12 hourly, creatinine clearance 10–50 ml/min 2 million units 24 hourly and creatinine clearance less than 10 ml/min 2 million units 36 hourly.

Tobramycin was given in doses of 5–6 mg/kg daily in patients with normal renal function. In patients with a creatinine clearance of less than 60 ml/minute, an initial dose of 3–4 mg/kg was administered with further dosing according to tobramycin plasma concentrations.

Efficacy outcomes assessed were microbiological clearance and death while in ICU. Microbiological clearance was defined as two or more consecutive negative cultures for *A. baumannii *from all sites sampled, within 10 days of initiation of the antimicrobial, with no subsequent positive cultures. The time to clearance was calculated as the time interval between antimicrobial initiation and the first negative culture.

The primary safety outcome assessed was the absolute increase in creatinine from baseline (the day before the antimicrobial was commenced) to the highest subsequent recorded value within 10 days of initiation of the antimicrobial. Participants receiving haemodialysis at the time antimicrobial therapy was first administered were excluded from this analysis. The proportion of participants in each group who increased their serum creatinine to greater than 50% above the upper limit of normal of the local reference values (100 μmol/ml in females, 120 μmol/ml males) was compared. All other adverse effects reported in the patient clinical records were recorded.

The study was approved by the University of Cape Town Health Science Faculty Research Ethics Committee.

### Statistical analysis

Data analysis was performed using Intercooled STATA™ version 8.2 (Statacorp, College Station, Texas). Continuous variables were summarised using means and standard deviations if normally distributed, and medians and ranges if not normally distributed. 95% confidence intervals (CIs) or interquartile ranges (IQRs) were calculated for all summary statistics and parameter estimates. Between-group comparisons of continuous data were performed using a Student's t test if normally distributed, and the Wilcoxon rank sum test if not normally distributed. Proportions were compared using a two sample test of proportions when the observed frequencies were ≥ 5 in each group and the Fisher's exact test if the expected frequencies were < 5. Kaplan-Meier curves were plotted for time to microbiological clearance and time to death while in ICU, and survival curves compared with a log-rank test. Patients were censored when they were discharged from ICU for the analysis of death and for death in the case of microbiological clearance. For all statistical analyses, a p value of less than 0.05 was regarded as sufficient evidence to reject the null hypothesis.

## Results

The baseline characteristics in the colistin and tobramycin groups were similar with respect to age, APACHE score at ICU admission and sites of isolation of *A. baumannii *(Table 1). The median baseline serum creatinine was higher in the colistin group (76 μmol/L) than in the tobramycin group (56 μmol/L), but this difference was not statistically significant (p = 0.08) Three patients in the colistin group and 5 in the tobramycin group were on haemodialyis at the time the antimicrobial was commenced and these proportions were not significantly different (Fishers exact test p = 0.7). Patients received a median of 8 days of colistin (interquartile range (IQR) 5 to 13) and 7 days of tobramycin (IQR 6 to 10) (p = 0.72).

**Table 1:** Baseline patient characteristics

	Colistin (n = 32)	Tobramycin (n = 32)	P value
Number of patients with chronic diseases at the time of admission (%)	9 (28)	12 (37.5)	0.65
Median duration of treatment (IQR)	8 (5 to 13)	7 (6 to 10)	0.72
Mean age in years (± SD)	43.5 (± 15.6)	45.6 (± 18.2)	0.69‡
Mean APACHE score at ICU admission (± SD)	14.4* (± 5.1)	14.8** (± 5.4)	0.77‡
Dialysis at baseline (%)	3 (9.4)	5 (15.6)	0.60†
Median baseline creatinine (IQR) umol/L	76 (53 to 128)	56 (45 to 91)	0.08
Site of *A. baumannii *infection:			
Bloodstream infection	10 (31%)	10 (31%)	1.00▫
Sputum/tracheal aspirate	22 (72%)	28 (88%)	0.13†
Wound pus swab	6 (18.8%)	11 (34.4%)	0.75▫
Cerebrospinal fluid	1 (3.1%)	0	NA
Central venous catheter tip	10 (31%)	9 (28%)	0.44▫
Urine	5 (15.6%)	4 (12.5%)	1.00†

*data available for 25 participants **data available for 21 participants † Fisher's Exact Test ‡ Two sample t- test • Wilcoxon rank sum test ▫ Two sample test of proportion

Either agent at our hospital is commenced after a review of the case by a clinical microbiologist. In addition, all, except one subject in the each group, had at least one feature to support the use of antimicrobial therapy for their *A. baumannii *documented in their records. Twenty patients (62.5%) in the colistin group and 22 (68.75%) patients in the tobramycin group met the criteria for sepsis (culture-positivity in conjunction with 2 or more components of the Systemic Inflammatory Response Syndrome) [10] at the time of initiation of the antimicrobial.

All the isolates in the colistin group were resistant to all antimicrobials tested, except colistin. All the isolates in the tobramycin group were susceptible to tobramycin. In 17 of the tobramycin cases (53%), the organism was only susceptible to tobramycin and colistin. Carbapenem resistance was present in 24 (75%) of the tobramycin group.

**Figure 2:**
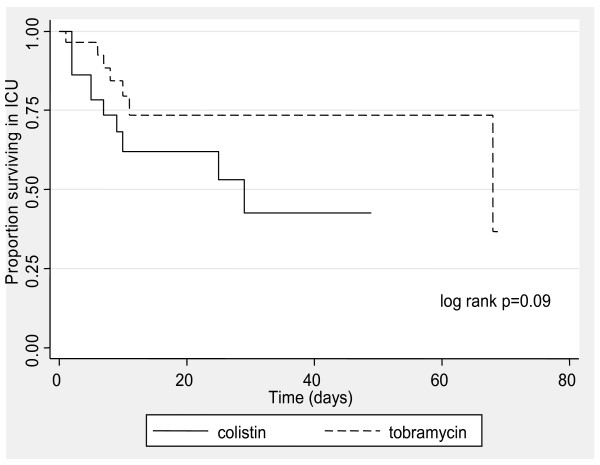
**Kaplan- Meier ICU survival curves by antibiotic**.

The median length of ICU stay after initiation of the antimicrobial was 6 days (IQR 4 to 21) in the colistin group and 9 days (IQR 4 to 21) in the tobramycin group (p = 0.06). Eleven colistin-treated patients (34.4%) and 7 tobramycin-treated patients (21.9%) died in ICU (p = 0.54). There was no significant difference in time to death in ICU by Kaplan-Meier survival analysis (log rank p = 0.09) (figure 2). The hazard ratio for ICU survival in patients treated with colistin compared with tobramycin was 0.44 (95% CI 0.16 to 1.19).

**Figure 3:**
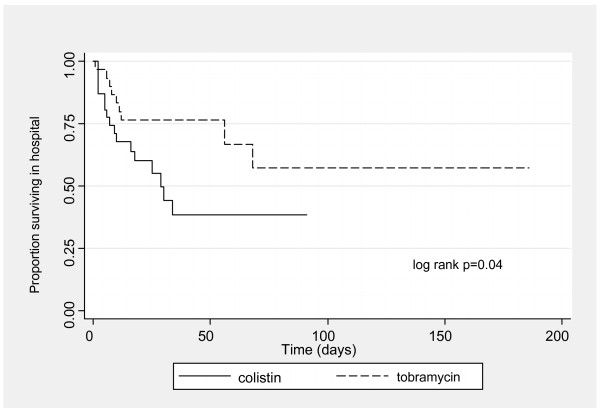
**Kaplan- Meier curves by antimicrobial for total in-hospital survival**.

The median length of total hospital stay after initiation of the antimicrobial was 18 days (IQR 7 to 34) in the colistin group and 27 days (IQR 12 to 56) in the tobramycin group (p = 0.08) Sixteen patients treated with colistin (50%) and 9 patients treated with tobramycin (28.1%) died in hospital. The hazard ratio for total in-hospital survival in patients treated with colistin compared to tobramycin was 0.43 (95% CI 0.19 to 0.99) (logrank p = 0.04) (see figure 3).

**Figure 4:**
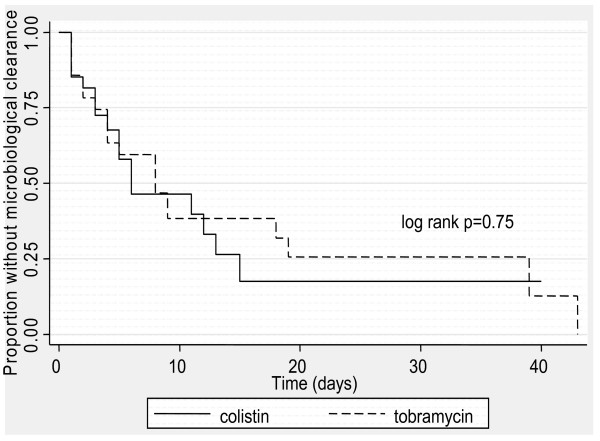
**Kaplan- Meier curves for microbiological clearance by antimicrobial**.

Bacteriological eradication was documented in 50% of patients treated with colistin and 55% treated with tobramycin (p = 0.79). The median time to clearance of A baumannii was 3 days for colistin and 4 days for tobramycin (p = 0.46). There was no significant difference in time to microbiological clearance by Kaplan-Meier analysis (figure 4) between the colistin and tobramycin groups (log rank p = 0.75). The hazard ratio for microbiological persistence in patients treated with colistin compared with tobramycin was 0.90 (95% CI 0.46 to 1.76).

There was a modest increase in creatinine from baseline to highest recorded within 10 days of antimicrobial commencement in both groups, with a median increase of 28 μmol/mL (IQR 11 to 135) in those on colistin and 17 μmol/mL (IQR 6 to 22) in those on tobramycin (Wilcoxon rank sum test p = 0.11). The geometric mean change in creatinine in the colistin group was 42 μmol/L (95% CI 24.4 to 74.9) and 19.6 μmol/L (12.1 to 31.6) in the tobramycin group. Only one participant in the cohort required initiation of haemodialysis after antimicrobial therapy was commenced, a male patient with a baseline serum creatinine of 118 μmol/mL 6 days after colistin initiation. The proportion of patients with normal renal function at baseline who increased their creatinine concentrations to greater than 50% above the upper limit of normal were similar in the two groups: 2 of 23 (8.7%) in the tobramycin group and 4 of 21 (19%) in the colistin group (Fishers exact test p = 0.67). No other adverse effects were documented.

Ten patients in each group had bloodstream infections. Colistin was used for a mean of 13 days (IQR 2 to 12.5) and tobramycin 7.5 days (IQR 3.5 to 11.5) for bloodstream infections (p = 0.25). Five patients in the tobramycin group (50%) and 3 patients (30%) in the tobramycin group with bloodstream infections died in ICU (p = 0.58).

Six patients with bloodstream infections in each group (60%) were demonstrated to have achieved sustained microbiological clearance.

## Discussion

We found no significant differences in ICU survival, time to microbiological clearance and elevations in serum creatinine between the tobramycin and colistin groups. These findings are encouraging, considering the uncertainties regarding the optimal dose of colistin to maximise efficacy while avoiding toxicity. Patients treated with colistin had higher in-hospital mortality; however we feel that in-ICU mortality is the more important measure as the ICU admission was the time period within which patients were exposed to colistin or tobramycin.

Two uncontrolled studies reported an incidence of nephrotoxicity associated with colistin usage of 14% [5,6]. However, the absence of a comparator arm in these studies makes it difficult to distinguish the nephrotoxicity induced by colistin from the effect of sepsis, hypotension and other potentially nephrotoxic drugs used in ICU.

Several studies have compared colistin with the carbapenems in ICU patients, and found that the mortality and nephrotoxicity rates were not different in the colistin and carbapenem groups [12-14]. However, in our intensive care units, in common with many other centres, an increasing proportion of *A. baumannii *isolates are resistant to the carbapenems. Seventy five percent of isolates in the tobramycin group in our study were carbapenem-resistant. Because of increasing carbapemen resistance, *A baumannii *infections in our institution are likely to be treated with either the aminoglycoside tobramycin, or colistin. To our knowledge there has been no study which directly compared aminoglycoside monotherapy with colistin. In one comparative study, 48% of patients in the non-colistin group received an aminoglycoside (in combination with a carbapenem, piperacillin-tazobactam or a quinolone) [13]. These investigators found no significant differences in mortality, the mean increase in creatinine or rates of microbiological persistence at day 7 between colistin-exposed and non-exposed patients [15].

We compared colistin with tobramycin. In a study of once daily administration of gentamicin and tobramycin in ICU patients, 14% of participants were reported to have a rise in creatinine of 45 μmol/mL or more [16]. Although a modest increase in creatinine concentrations occurred in both groups in our study, the magnitude was similar for patients administered either colistin or tobramycin. The cohort of ICU patients was heterogenous and at high risk for the development of renal failure. Our results suggest that colistin, when used in critically ill patients at relatively high doses, is no more nephrotoxic than tobramycin.

Our study has several limitations. The study population is heterogenous, as we included all intensive care unit patients with *A. baumannii *infections from any site. Clinical patient information was gathered retrospectively, resulting in some missing data and we were not able to control for inter-observer variability. The sample size is small, however as colistin is only prescribed when there are no other available antimicrobials and sample sizes in other studies have been similar. The primary outcome was the change in creatinine concentration in those patients who were not receiving dialysis. There was a high degree of variability in the change in creatinine in the colistin group and the 95% confidence intervals for the geometric mean are wide. It is therefore possible that we failed to demonstrate a difference between the colistin and tobramycin groups because of our limited sample size. In the ICU setting, sepsis, hypotension and the use of other nephrotoxic drugs contribute to impairment of renal function. We are not able to exclude the confounding impact of these variables.

## Conclusion

Our data suggests that colistin is not significantly different to tobramycin in terms of efficacy or nephrotoxicity and that it is an acceptable treatment of *A. baumanii *infections when the organism is resistant to other available antimicrobials.

## Competing interests

The authors declare that they have no competing interests.

## Authors' contributions

RG contributed to the design of the study, the acquisition and analysis of data and preparation of the manuscript. CB contributed to study design, the acquisition of data and preparation of the manuscript. RZS was involved in the study design and the preparation of the manuscript. KC contributed to the analysis of the data and the preparation of the manuscript. GM contributed to the design of the study, data analysis, the preparation of the manuscript, and supervised the study. All authors read and approved the final manuscript.

## Pre-publication history

The pre-publication history for this paper can be accessed here:

http://www.biomedcentral.com/1471-2334/9/26/prepub

## References

[B1] Li J Nation R Milne R Turnidge J Coulthard K Evaluation of colistin as an agent against multi-resistant Gram-negative bacteria Int J Antimicrob Agents 2005 25 11 25 10.1016/j.ijantimicag.2004.10.001 15620821

[B2] Berlana D Llop J Fort E Badia M Jodar R Use of colistin in the treatment of multiple- drug -resistant gram- negative infections Am J Health – Syst Pharm 2005 62 39 47 10.1093/ajhp/62.1.39 15658071

[B3] Jian Li Rayner C Nation R Owen R Spelman D Tan K Liolios L Heteroresistance to Colistin in Multidrug-Resistant *Acinetobacter Baumannii* Antimicrob Agents Chemother 2006 50 2946 50 16940086 10.1128/AAC.00103-06 PMC1563544

[B4] Li J Nation R Turnidge J Milne R Coulthard K Rayner C Paterson D Colistin: the re-emerging antibiotic for multi-drug resistant Gram-negative bacterial infections Lancet Infect Dis 2006 6 589 601 10.1016/S1473-3099(06)70580-1 16931410

[B5] Falagas M Fragoulis K Kasiakou S Sermaidis G Michalopoulos A Nephrotoxicity of intravenous colistin: a prospective evaluation Int J Antimicrob Agents 2005 26 504 7 10.1016/j.ijantimicag.2005.09.004 16280245

[B6] Markou N Apostolakos H Koumoudiou C Athanasiou M Koutsoukou A Alamanos I Intravenous colistin in the treatment of sepsis from multiresistant Gram-negative bacilli in critically ill patients Critical Care 2003 7 R78 83 12974973 10.1186/cc2358 PMC270720

[B7] Falagas E Kasiakou S Toxicity of polymyxins: a systematic review of the evidence from old and recent studies Crit Care 2006 10 1 R27 16507149 10.1186/cc3995 PMC1550802

[B8] Munoz-Price L Weinstein R Acinetobacter infection N Engl J Med 358 1271 81 10.1056/NEJMra070741 18354105

[B9] Jackson W Shorr A Update in ventilator-associated pneumonia Curr Opin Anaesthesiol 2006 19 117 21 10.1097/01.aco.0000192770.01904.dd 16552216

[B10] American College of Chest Physicians/Society of Critical Care Medicine Consensus Conference: definitions for sepsis and organ failure and guidelines for the use of innovative therapies in sepsis Crit Care Med 1992 6 864 74 1597042

[B11] Colimycine [package insert], Bellon, France: Aventis 1997

[B12] Rios F Luna C Maskin B Valiente A Lloria M Gando S Ventilator-associated pneumonia due to colistin susceptible-only microorganisms Eur Resp J 2007 30 307 313 10.1183/09031936.00156906 17504791

[B13] Kallel H Hergafi L Bahloul H Hakim A Dammak H Chelly H Safety and efficacy of colistin compared with imipenem in the treatment of ventilator- associated pneumonia: a matched case-control study Intensive Care Med 2007 33 1162 7 10.1007/s00134-007-0675-2 17530220

[B14] Garnacho-Montero J Ortiz-Leyba C Jiménez-Jiménez A Barrero-Almodóvar L Garcia-Garmendia L Bernabeu Wittell M Treatment of Multidrug-Resistant *Acinetobacter Baumannii *Ventilator-Associated Pneumonia (VAP) with Intravenous Colistin: A Comparison with Imipenem-Susceptible VAP Clin Infect Dis 2003 36 1111 8 10.1086/374337 12715304

[B15] Reina R Estenssoro E Saenz G Canales H Gonzalvo R Vidal G Safety and Efficacy of Colistin in *Acinetobacter *and *Pseudomonas *infections: a prospective cohort study Intensive Care Med 2005 31 1058 65 10.1007/s00134-005-2691-4 15983759

[B16] Bjuik S Mouton J Gyssens I Verbrugh H Bruining H Experience with a once-daily dosing program of aminoglycosides in critically ill patients Intensive Care Med 2002 28 936 942 10.1007/s00134-002-1313-7 12122533

